# Supplementary material for: Application of Micron-Sized Zero-Valent Iron (ZVI) for Decomposition of Industrial Amaranth Dyes
Source: Materials (Basel). 2023 Feb 11;16(4):1523. doi: 10.3390/ma16041523 (PMC9967265; doi:10.3390/ma16041523)
Supplement: Supplementary file 1 [file materials-16-01523-s001.zip › materials-2129550-supplementary.pdf]

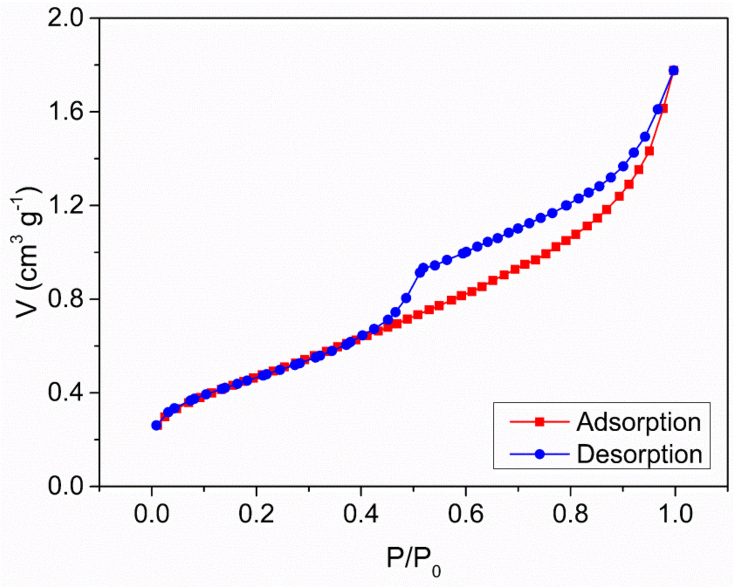

Figure S1. Nitrogen adsorption–desorption isotherms.

Table S1. BET-specific surface area results for iron compounds.

**Material**	**Surface area (m^2 g^{-1})**	**Reference**
ZVI	1.711	Our work
Hematite	12.71	[27]
Goethite	2.13	[28]
Magnetite	5.60	[29]
